# The evidence base for pilonidal sinus surgery is the pits

**DOI:** 10.1007/s10151-019-02116-5

**Published:** 2019-11-21

**Authors:** S. R. Brown, J. N. Lund

**Affiliations:** 1 University of Sheffield, Sheffield, UK; 2 University of Nottingham, Nottingham, UK

## Abridged abstract [1]

*Background* Pilonidal sinus arises in the hair follicles in the buttock cleft. The estimated incidence is 26 per 100,000, people, affecting men twice as often as women. These chronic discharging wounds cause pain and impact upon quality of life. Surgical strategies centre on excision of the sinus tracts followed by primary closure and healing by primary intention or leaving the wound open to heal by secondary intention. There is uncertainty as to whether open or closed surgical management is more effective.

*Objectives* To determine the relative effects of open compared with closed surgical treatment for pilonidal sinus on the outcomes of time to healing, infection and recurrence rate.

*Selection criteria* All randomised controlled trials (RCTs) comparing open with closed surgical treatment for pilonidal sinus. Exclusion criteria were: non‐RCTs, children aged younger than 14 years and studies of pilonidal abscess.

*Main results* For this update, 8 additional trials were identified giving a total of 26 included studies (*n* = 2530). Seventeen studies compared open wound healing with surgical closure. Healing times were faster after surgical closure compared with open healing. Surgical site infection (SSI) rates did not differ between treatments; recurrence rates were lower in open healing than with primary closure (RR 0.60, 95% CI 0.42–0.87). Six studies compared surgical midline with off‐midline closure. Healing times were faster after off midline closure (MD 5.4 days, 95% CI 2.3–8.5). SSI rates were higher after midline closure (RR 3.72, 95% CI 1.86–7.42) and recurrence rates were higher after midline closure (Peto OR 4.54, 95% CI 2.30–8.96).

*Authors’ conclusions* No clear benefit was shown for open healing over surgical closure. A clear benefit was shown in favour of off midline rather than midline wound closure. When closure of pilonidal sinuses is the desired surgical option, off‐midline closure should be the standard management.

This Cochrane review [1] supports a generally held view by pilonidal surgery experts that, when excision and closure is carried out, closure should avoid the midline if optimal outcomes are to be achieved. However, despite this evidence, primary midline closure is still commonly carried out [2]. In addition, subsequent consensus and guideline publications suggest excision of disease and leaving the wound open results in prolonged recovery compared with closure techniques [2, 3] and yet the ‘leave open’ technique is the most commonly performed operation in the UK and other European countries [2, 4].

Why should this be? Reasons are potentially twofold

- There is a lack of experience and/or skill or interest in dealing with pilonidal disease by most surgeons.

Simple excision with or without primary closure is easy and quick. Alternative techniques such as Karydakis, Bascom’s cleft closure and flaps are more complicated and do not really fall into the remit particularly of a colorectal specialist. Operations for PSD may be delegated to juniors or the condition is just not of sufficient interest to many surgeons to keep up to date with the literature or learn new techniques.

- The evidence is not believable.

There is some basis to this perception. The evidence on the whole for pilonidal disease surgery is poor, with the majority of publications being case series or non-randomised comparative trials from single-centre institutions, with the inherent bias that such studies bring. Nearly 90% of over 250 published studies in the 10 years since the AL-Khamis review are of this type and arguably should be ignored when reaching any consensus. Even the data included in this Cochrane review have faults, and these faults persist in subsequent and current reports. These faults include:

- *Lack of a widely accepted and validated classification system.* Pilonidal disease can vary from a simple asymptomatic pit through to extensive disease with multiple midline pits and lateral extensions, sometimes accompanied by marked scarring and deformity from previous failed interventions. Unless there is an objective way that PSD can be stratified on randomisation or assurance that the baseline of each group is the same, any randomised controlled trial will be at great risk of being invalid.
- *Single-centre studies* The studies included in this review were mainly single centre in design with procedures likely to have been performed by advocates and pioneers of the intervention. Of approximately 35 RCTs published on pilonidal sinus surgery since this review, over 90% are single centre. Techniques need to be generalisable and real-world outcomes can only be estimated in multicentre trials.
- *Multiple interventions and comparators* Pilonidal surgery is a classic example of a disease where there is no obvious gold-standard treatment. As a consequence, multiple interventions exist. There is even variation within the recognised techniques and standardisation is poor. Whilst this review has been able to group the trials into two general groups, allowing some useful practical conclusions to be drawn (i.e. avoid the midline closure), the literature makes more definitive guidance almost impossible to achieve. In RCTs published since 2010, around 16 different interventions are compared with around 14 different controls. This makes meaningful meta-analysis extremely challenging and comparison of ‘apples with pears’ and, like all meta analyses, need to be interpreted with care [5]. Moreover, comparing obsolete with current best practice is unhelpful and a waste of resources. For instance, an RCT of a novel intervention is meaningless if the comparator is excision and primary midline closure or even leave open, interventions considered inferior by the pilonidal expert community. Around 2/3 of the studies with higher quality design performed in the last 10 years use invalid comparators, and as such lose much of their power to inform practice.
- *Lack of an adequate sample size* Trial numbers for the most recent studies vary from 19 to 800, with a median of around 70 patients for each arm of a comparative trial. Many studies have no evidence of any sample size calculation and in others, the details are vague, such that 75% of these trials can be considered inadequately powered. Few account for dropout even though this is likely to be high given the young mainly male and often mobile population. Several studies suggest a 100% follow-up even up to 5 years which is astoundingly complete!
- *Selection of inappropriate outcome measures* The selection of an appropriate outcome measure is perhaps the main criticism of any current pilonidal research. Of the RCTs published since the 2010 Cochrane review most do not state a primary outcome. Of those that do, outcomes vary from operative time, wound healing, wound closure, infection rates, return to normal activities/work, quality of life and, of course, recurrence.

The Cochrane review details healing as one of the main outcomes. Even this superficially straightforward endpoint leaves many areas that need to be clarified that are not addressed on the whole in studies using it as a primary endpoint. These ambiguities include: when does healing occur? How was it assessed? Did the patients truly and accurately define the day of healing? How did they know? Was there clinical assessment on a daily basis? The accuracy of such an outcome measure has to be questioned and methods to measure it more precisely developed. The same is true for recurrence, another commonly used primary outcome and reported in virtually every paper in the Cochrane review. Is recurrence persistent non-healing or development of new disease after clinical evidence of healing after the study procedure, or indeed both? How long is follow-up necessary before recurrence can be dismissed? Follow-up varied from 6 months to more than 3 years in RCTs reported after 2010.

- *Outcome measures not relevant to patients* Pilonidal sinus disease occurs in young adults at an age where body image is more important than at other stages of life, when relationships are formed and attendance at study or work is crucial to progression in life. In this group, long-term healing may not be the thing they want to achieve at all costs, and especially not at the cost of disfiguring scaring, packing or time away from normal activities. If one intervention can be done as an office procedure under local anaesthetic with rapid recovery and minimal post-operative pain but a 25% chance of recurrence, do patients value that more than an operation that requires a general anaesthetic, a stay in hospital, a drain and weeks recovering and/or a potential need for regular nursing input for dressings but a recurrence rate of 1–2% (although the real-world recurrence rate may well be much higher)? For PSD in children, treatments avoiding time off school is probably paramount.
- *Cost effectiveness rarely considered* The Cochrane review correctly suggested that meaningful research should also consider cost effectiveness as an outcome. Some procedures that require intensive post-operative health care involvement (e.g. regular dressings or packing) will have significant healthcare staff and consumable costs in addition to societal costs, including time off work, in a young, active working population. Increased equipment costs for interventions such as endoscopic pilonidal sinus treatment (EpSiT) and laser therapy or consumable costs such as fibrin glue must be also be included in the cost effectiveness calculations.

It is clear that we need better evidence on how best to treat this unpleasant condition that has a significant impact on the young lives of sufferers. Future trials should address the deficiencies in design outlined above. A standardised and accepted classification system should be developed to minimise bias, allow readers to know exactly what kind of disease the intervention was treating and facilitate comparison of trial with trial and secondary analysis of results. Trials should be multicentric (as suggested in the review) to maximise the chance of successful in trial treatments being generalisable. Multicentre trials will also take into account inter-surgeon variation in technique, although such trials should seek to standardise technique by consensus as part of their design. Comparison should be between front running interventions and comparison should not be made with redundant, if commonly used, techniques. Niche and novel interventions can still be explored but according to idea, development, exploration, assessment, long-term study (IDEAL) framework criteria [6]. Studies should ideally include health economic assessment, which incorporates evaluation of cost to primary and secondary care and also, if possible, cost to society in terms of time off work and similar metrics. Finally, and probably most importantly, it is patients who suffer from pilonidal sinus disease, and it is them who all too frequently have to suffer a treatment that may be worse than the disease. More work is needed on what patients’ value in terms of outcomes rather than a focus on what the surgeon perceives to be the most appropriate outcome measure. We have not moved the evidence base forward in any meaningful way since the Al-Khamis Cochrane review in 2010. Pilonidal sinus disease has a significant impact on quality of life in young people and it is for the patients that we should do better, designing modern robust trials to provide high-quality evidence to inform decision making. Ask yourself if you would want to have a large hole cut in your natal cleft, left to heal by secondary intention over months?: if it’s not suitable for you, it’s not suitable for patients with pilonidal sinus disease. Let’s provide the evidence we need to move us out of the dark ages.
